# Human Metapneumovirus, Australia, 2001–2004

**DOI:** 10.3201/eid1208.051239

**Published:** 2006-08

**Authors:** Theo P. Sloots, Ian M. Mackay, Seweryn Bialasiewicz, Kevin C. Jacob, Emily McQueen, Gerald B. Harnett, David J. Siebert, I. Brent Masters, Paul R. Young, Michael D. Nissen

**Affiliations:** * Royal Children's Hospital and Health Service District, Brisbane, Queensland, Australia;; † Clinical Medical Virology Centre at University of Queensland, Brisbane, Queensland, Australia;; ‡ University of Queensland, Brisbane, Queensland, Australia;; § Queensland Health Pathology Service, Brisbane, Queensland, Australia;; ¶ PathWest Laboratory Medicine, Perth, Western Australia, Australia

**Keywords:** human metapneumovirus, acute respiratory infection, respiratory viruses, epidemiology, genotyping, molecular, clinical, pediatric

## Abstract

We examined 10,025 respiratory samples collected for 4 years (2001–2004) and found a 7.1% average annual incidence of human metapneumovirus. The epidemic peak of infection was late winter to spring, and genotyping showed a change in predominant viral genotype in 3 of the 4 years.

Human metapneumovirus (HMPV) is now recognized as a substantial cause of acute respiratory infection, particularly in children; it has phenotypic and clinical characteristics that are similar to those of respiratory syncytial virus (RSV) (*1**,**2*). HMPV is ubiquitous; it infects most children at an early age, and distinct epidemic peaks are reported in the winter months (*1**,**3**–**6*). However, many of theses studies were conducted in the Northern Hemisphere and involved samples collected during a relatively short period; few data exist from extended studies over several years that involve populations in other parts of the world.

Although 2 main HMPV types are recognized (A and B) (*1**,**3**,**7*), each with 2 subtypes (A1, A2; B1, B2) (*8*), the extent of genetic variation of circulating HMPV subtypes over time has not been extensively examined. We sought to determine the incidence and seasonal distribution of HMPV in an Australian population from 2001 to 2004 and to establish the pattern of genotype distribution during those 4 years by examining the genetic variability of the P gene in 640 of 707 HMPV-positive samples.

## The Study

The necessary ethics approval for this study was obtained from the Royal Children's Hospital ethics committee. We collected nasopharyngeal aspirate (NPA) specimens from January 2001 through December 2004 from patients with acute lower respiratory tract infection in Queensland, Australia. Patients were from 3 months to 93 years of age (mean 8.2 years, median 1.37 years), and 78.1% of specimens were from children <5 years of age. Nucleic acids were extracted from 0.2 mL of each NPA specimen by using the High Pure Viral Nucleic Acid kit (Roche Diagnostics, Mannheim, Germany), according to the manufacturer's instructions. Extracts were analyzed for HMPV sequences by reverse transcriptase PCR (*9*). For samples collected during 2001 and 2002, other viral respiratory pathogens were detected by using a direct fluorescent antibody assay (DFA) in combination with a culture-augmented DFA method (*10*). For samples collected in 2003 and 2004, these pathogens were detected by multiplex PCR (*10*).

**Figure 1:**
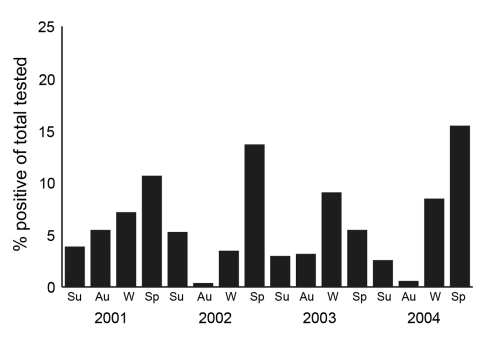
Seasonal incidence of human metapneumovirus, Queensland, Australia, 2001–2004. Su, summer (December–February); Au, autumn (March–May); W, winter (June–August); Sp, spring (September–November).

Of 10,025 NPA specimens tested, 707 were positive for HMPV, for an overall incidence of 7.1% during the 4 years. The youngest HMPV-positive patient was 4 months old, and the oldest was 79 years. In children (<18 years of age) the incidence of virus was 7.4%, and 91.9% of HMPV-positive children were <5 years of age. The seasonal distribution of HMPV infection showed a distinctive pattern for each of the 4 years studied (Figure 1). In 2001, HMPV showed broad seasonal activity; incidence was >5% in 3 consecutive seasons (autumn, winter, and spring) and peaked at 10.6% in the spring (September–November). In 2002 and 2004, most HMPV activity was in spring, (incidence of 13.6% and 15.4%, respectively), with little evidence during autumn (March–May). In 2003, the peak incidence of 9.0% occurred in winter (June–August) and persisted into spring (5.4%). In all years, virus was present well into summer (December–February), with an incidence ranging from 2.5% to 5.2%. On examination of those samples collected in 2003 and 2004, which were all previously analyzed for common respiratory viruses by PCR, HMPV was the most frequently detected respiratory virus in children during the spring of each year. Expressed as an annual average over the 4 years studied, the predominant viral pathogen was RSV (9.2%), followed by HMPV (7.1%), influenza A (3.5%), parainfluenza virus 3 (2.3%), and adenovirus (1.3%). In 6.8% of HMPV-positive cases, evidence of co-infection with another respiratory virus was seen; 20 patients were concurrently infected with an adenovirus, 10 with influenza A virus, 8 with RSV, 9 with parainfluenza virus 3, and 1 with parainfluenza virus 2.

**Figure 2:**
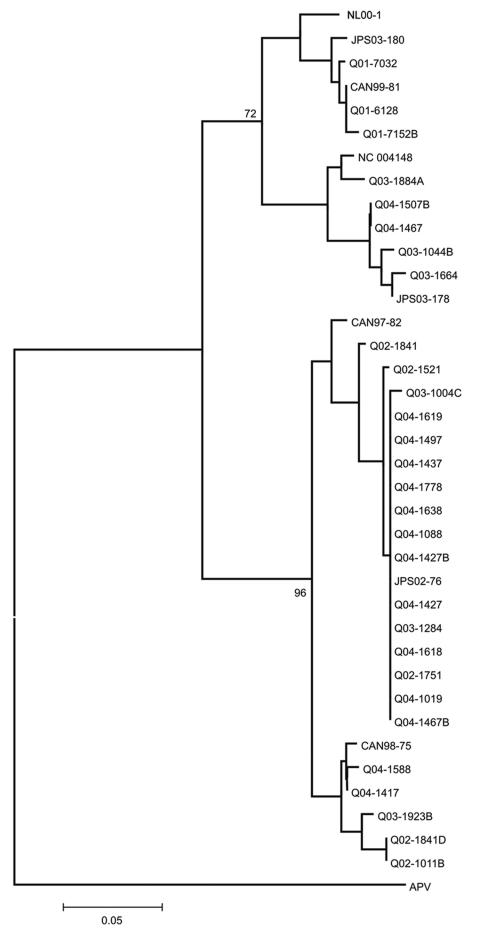
Phylogenetic analysis of the 182-nucleotide fragment of the phosphoprotein gene fragment of human metapneumovirus detected in respiratory samples collected in Australia. Sequences of avian pneumovirus (APV) type C (GenBank accession nos. AF176590 and AF176591) were used as outgroups to root the tree. Nucleotide sequences were aligned by using BioEdit version 7.0.0 and were subjected to neighbor-joining analysis with MEGA version 3.0 with 500 random bootstraps. CAN and NC, Canada; JPS, Japan; NL00–1, the Netherlands; Q, Queensland, Australia.

Amplification products generated directly from 640 HMPV-positive NPA specimens were genotyped as previously described (*11*) (GenBank accession nos. DQ112292–DQ112320 and DQ121378–DQ121384). Data showed that all 4 viral subtypes cocirculated during each of the 4 years studied (Table 1, Figure 2). However, a different subtype predominated during 3 of the 4 years: HMPV subtype A1 was dominant in 2001, subtype A2 in 2002 and 2003, and subtype B1 in 2004 (Table 1).

**Table 1:** Distribution of human metapneumovirus (HMPV) subtypes in Queensland, Australia, 2001–2004

Year	Total samples tested	HMPV subtype			
		A1	A2	B1	B2
2001	59	58	24	8	10
2002	122	20	51	12	17
2003	189	10	36	30	24
2004	270	1	23	59	17

Clinical records from 273 patients who were positive for HMPV were scrutinized, and data describing clinical features and length of hospital stay were recorded. Of these patients, 203 (74.4%) were admitted to hospital with a median length of admission of 3 days and a mean of 6.5 days. The predominant clinical features were cough (63%), rhinorrhea (61%), respiratory crackles/crepitations (60%), and fever (57%) (Table 2). Ninety (33.9%) of the 273 patients had a chest radiograph, and 77 (85.6%) patients showed bilateral parahilar peribronchial infiltrates consistent with a lower respiratory tract infection. Disease severity of the 273 HMPV-positive patients was classified as mild (46.8%), moderate (42.5%), and severe (10.7%), based on the use of supplemental oxygen and fluids and length of hospital stay.

**Table 2:** Signs and symptoms noted with human metapneumovirus infection (N = 273)

Clinical feature	%
Cough	63
Rhinorrhea	61
Crackles/crepitations	60
Fever	57
Respiratory distress	48
Anorexia	45
Vomiting	39
Wheezing	38
Irritability	31
Tachypnea	30
Lethargy	26
Pharyngitis/tonsillitis	24
Dry mouth	23
Diarrhea	18
Otitis media	15
Noisy breathing	14
Rash	10
Conjunctivitis	7
Cyanosis	4
Apnea	2
Hoarseness	1

## Conclusions

This study of HMPV infection is the largest so far reported. The results of recent, similar studies suggested that peak periods of infection with HMPV predominate during winter in the Northern Hemisphere. However, this finding has not been extensively examined over an extended period with a large, continuous sample. Our study found that the peak period of HMPV infection in Queensland, Australia, occurs predominantly in spring (August–October) but that HMPV can be detected in every month. This finding suggests that HMPV activity, like RSV activity, occurs in the community throughout the year, and peaks of infection are a result of seasonal environmental factors.

Although RSV predominated in all years, HMPV was the second most frequently detected virus in each year studied. The low rate of co-infection of HMPV with other respiratory viruses (including RSV) suggests that co-infection may not be common in our community. When analyzing disease severity in this sample with a 2-sided test of proportions, we saw no significant difference between patients with a co-infection and those without. Therefore, our data did not support the suggestion by others that co-infection of HMPV with RSV or other viral respiratory pathogens is a risk factor for severe disease (*6*).

The shift in predominant HMPV genotype observed in this study was similar to those reported for RSV and influenza viruses (*6*) and can be attributed to changes in immunity of the population in response to antigenic differences between the predominant circulating strains (*12**,**13*). However, a relationship between genotype and disease severity, as previously established for RSV (*14**,**15*), did not appear to apply for HMPV, but we plan to examine this relationship further.

The clinical features associated with HMPV infection in this study were not sufficiently distinctive to clinically differentiate it from other respiratory viral infections in children, particularly those attributed to RSV. In addition, few patients (10.6%) had severe disease, but most (76%) were sufficiently ill to be admitted and treated in the hospital for >3 days, which represents a substantial amount in healthcare costs.

Finally, this comprehensive study, conducted for 48 months, is the first one aimed at establishing an accurate estimate of the incidence and seasonal distribution of HMPV infection and to determine the genetic variation of HMPV circulating in our population. The clinical spectrum of infection in a substantial proportion of HMPV-positive patients has been described, and studies are continuing to fully elucidate the clinical effect of infection with this virus in our community.
